# The Relationship between Dissolution Behavior and the Toxicity of Silver Nanoparticles on Zebrafish Embryos in Different Ionic Environments

**DOI:** 10.3390/nano8090652

**Published:** 2018-08-23

**Authors:** Wang Sik Lee, Eungwang Kim, Hyun-Ju Cho, Taejoon Kang, Bongsoo Kim, Min Young Kim, Yong Sik Kim, Nam Woong Song, Jeong-Soo Lee, Jinyoung Jeong

**Affiliations:** 1 Hazards Monitoring Bionano Research Center, Korea Research Institute of Bioscience and Biotechnology, 125 Gwahak-ro, Yuseong-gu, Daejeon 34141, Korea; wang3026@kribb.re.kr (W.S.L.); alleles@kribb.re.kr (H.-J.C.); kangtaejoon@kribb.re.kr (T.K.); joody1006@naver.com (M.Y.K.); jsa1713@naver.com (Y.S.K.); jeongsoo@kribb.re.kr (J.-S.L.); 2 KRIBB School, University of Science and Technology, 125 Gwahak-ro, Yuseong-gu, Daejeon 34141, Korea; 3 Department of Chemistry, Korea Advanced Institute of Science and Technology, 291 Daehak-ro, Yuseong-gu, Daejeon 34141, Korea; kekgsm@kaist.ac.kr (E.K.); nanobio@kaist.ac.kr (B.K.); 4 Advanced Instrumentation Institute, Korea Research Institute of Standards and Science, 267 Gajeong-ro, Yuseong-gu, Daejeon 34113, Korea; nwsong@kriss.re.kr; 5 Dementia DTC R&D Convergence Program, Korea Institute of Science and Technology, 5 Hwarang-ro, 14-gil, Seongbuk-gu, Seoul 02792, Korea

**Keywords:** silver nanoparticle, dissolution behavior, zebrafish embryo, surface functionalization

## Abstract

A silver nanoparticle is one of the representative engineered nanomaterials with excellent optical, electrical, antibacterial properties. Silver nanoparticles are being increasingly used for medical products, water filters, and cosmetics, etc. However, silver nanoparticles are known to cause adverse effects on the ecosystem and human health. To utilize silver nanoparticles with minimized negative effects, it is important to understand the behavior of silver nanoparticles released to the environment. In this study, we compared toxicity behaviors of citrate-stabilized silver nanoparticles with polyethylene glycol coated silver nanoparticles in two different ionic environments, which are aquatic environments for developing zebrafish embryo. Depending on the composition of the ionic environment, citrate-stabilized silver nanoparticles and polyethylene glycol coated silver nanoparticles exhibited different behaviors in dissolution, aggregation, or precipitation, which governed the toxicity of silver nanoparticles on zebrafish embryos.

## 1. Introduction

Silver nanoparticles (AgNPs) are widely used nanomaterials for industrial and biomedical fields due to their unique physicochemical properties such as excellent conductivity, optical functions, and antibacterial capability [1,2,3,4]. However, the widespread use of AgNP results in an increasing amount of AgNPs being released into the environment, which can affect the health of humans and ecosystems [5,6]. Because AgNPs are sensitive to the surrounding environment, environmental transformations of AgNPs, such as aggregation, oxidation, or dissolution, frequently occur [7,8]. These transformations are attributed to the reduced thermodynamic stability of AgNPs by substances that constitute the aquatic environment, such as the amount of sulfides, chlorides, or organic matters, ionic strength, and pH [7,9]. Thus, understanding behaviors of AgNPs in an aquatic environment is important for evaluating the toxicity of AgNPs [10]. Among many factors, the chloride ion is closely related to the chemical transformation of AgNP depending on the concentration of Cl^−^. For example, Groh et al. suggested that the high concentration of the chloride ion may lead to formation of various dissolved silver-chloride complexes and influence the actual concentration of the Ag ion, which is toxic to the organisms [11]. They reported the silver ion-mediated toxicity of AgNP in a low chloride ion-containing medium, which minimizes the soluble chlorocomplex or formation of AgCl. However, it also showed the limitation of actual toxicity of AgNP, because the toxicity experiments were done with the bunch of zebrafish embryos (50 eggs/petri dish), not individuals. Therefore, it is necessary to investigate the relationship between the transformation of AgNPs and toxicity in individual zebrafish embryos in different ionic environments. 

The toxicities of AgNPs were evaluated in various animal models such as zebrafish (Danio rerio) [12,13], Caenorhabditis elegans [14], and Drosophila melanogaster [15]. In particular, zebrafish embryo is a suitable animal model due to its high fecundity, rapid embryonic development, cost effectiveness, and facile process, which allowed for a in vivo toxicity test to be performed [16]. Zebrafish embryo can provide various assessment methods to evaluate engineered nanomaterials (ENMs) toxicity such as mortality, hatching achievement, in vitro/in vivo imaging, and developmental malformation analysis of embryos, organ behavioral analysis, and immunotoxicity [17]. Thus, a toxicity study using zebrafish embryos provides intuitive experiments on the interaction between matter and the ecological environment.

**Figure 1:**
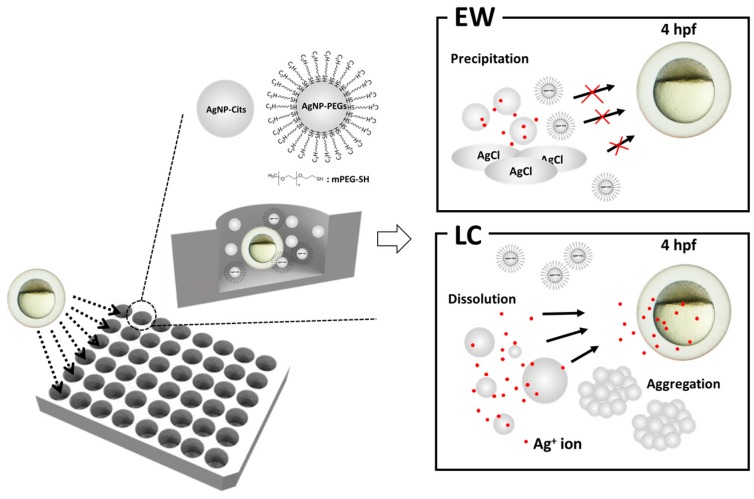
Schematic illustration of citrate-stabilized AgNPs (AgNPs-Cit) and polyethylene glycol-coated AgNPs (AgNPs-PEG) toxicity evaluations in different ionic environments.

In this study, we present the relationship between the dissolution behavior and toxicity of AgNPs in zebrafish embryo development environment. We hypothesized that the transformation of AgNPs will influence the toxicity of zebrafish embryo. To determine the behavior of AgNPs in ionic environments such as E3 egg water (EW) and low chloride (LC) medium, we analyzed the physicochemical properties of citrate-stabilized AgNPs (AgNPs-Cit) and polyethylene glycol (PEG)-coated AgNPs (AgNPs-PEG). We compared the toxicities of the AgNPs-Cit and AgNPs-PEG in different ionic environments using individual zebrafish embryos (Figure 1). 

## 2. Materials and Methods

### 2.1. Materials

Silver nitrate (AgNO_3_) and sodium citrate (Na_3_C_6_H_5_O_7_·5H_2_O) were purchased from Sigma-Aldrich (St. Louis, MO, USA). Methoxy polyethylene glycol sulfhydryl (mPEG-SH, 5000 Da) was purchased from SunBio (Gunpo-si, Gyeonggi-do, Korea). EW and LC media were prepared by dissolving sodium chloride (NaCl), calcium chloride (CaCl_2_), potassium chloride (KCl), magnesium sulfate (MgSO_4_), sodium phosphate monobasic (NaHPO_4_), potassium phosphate monobasic (KH_2_PO_4_), and sodium bicarbonate (NaHCO_3_) in 1 L of distilled water (DW) [11,18]. All reagents were purchased from Sigma-Aldrich (St. Louis, MO, USA), except sodium bicarbonate (NaHCO_3_). Sodium bicarbonate (NaHCO_3_) was purchased from DAEJUNG (Siheung-si, Gyunggi-do, Korea). Table 1 showed the composition of EW and LC medium.

### 2.2. Syntheses of AgNPs-Cit and AgNPs-PEG

AgNPs were synthesized from reduction method. AgNO_3_ of 15 mg was dissolved in 100 mL of DI water in Erlenmeyer flask and heated up to 100 °C with vigorous stirring. Sodium citrate solution (10 mg/mL) 20 mL was added to the solution at 100 °C and stirring for 10 min. After 10 min, the solution was cooled down to room temperature (RT). The resultant solution was filtered by 0.22 µm polyvinylidene difluoride (PVDF) membrane filter (Merck Millipore Ltd., Burlington, MA, USA). To prepare AgNPs-PEG, mPEG-SH (5000 Da, 1 mg/mL in DI water (SunBio, Gunpo-si, Gyeonggi-do, Korea)) 9 mL was mixed with the filtrate 36 mL and gentle stirring for 60 min. AgNP-PEG suspension was washed using Amicon ultra centrifugal filter (100 kDa, Merck Millipore Ltd., Burlington, MA, USA) at 4000 rpm for 15 min. After centrifugation, the solid was re-dispersed in DW. 

### 2.3. Characterizations of AgNPs-Cit and AgNPs-PEG

The size distributions and morphologies of AgNPs-Cit and AgNPs-PEG were analyzed using 200 kV field-emission transmission electron microscope (FE-TEM, JEM-2100F, JEOL LTD, Tokyo, Japan) To measure the FE-TEM, AgNPs-Cit and AgNPs-PEG (80 µg/mL) were sonicated for 10 min, and the supernatants were dropped onto a copper grid and dried at RT overnight. The behaviors of the particles were analyzed to the zeta potential, hydrodynamic size, and absorption spectra. The zeta potential was measured by Zeta-sizer (Nano ZS, Malvern Instruments Ltd., Malvern, WR, UK). Hydrodynamic size was measured by dynamic light scattering (DLS, Otsuka, Tokyo, Japan), and absorption spectra were UV-Visible spectroscopy (DU800, Beckman, Brea, CA, USA). AgNPs-Cit and AgNPs-PEG (5 µg/mL) were immersed in DW, EW, and LC media and analyzed. 

X-ray photoelectron spectroscopy (XPS) analyses were carried out using PHI 5000 VersaProbe (ULVAC-PHI, Osaka, Japan) with monochromatic Al K_α_ (1486.6 eV) radiation source. To prepare the samples, AgNPs were immersed in DW, EW, and LC for 1 day, then dropped and dried on silicon wafers.

### 2.4. Zebrafish Embryo Toxicity Test

Zebrafish embryos were obtained following previous literature, and all zebrafish experiments were performed in compliance with guidelines from the Korea Research Institute of Bioscience and Biotechnology (KRIBB) and approval by KRIBB-IACUC (approval No. KRIBB-AEC-17122). For mortality test, healthy zebrafish embryos at 4 h post-fertilization (hpf) were exposed to the AgNPs-Cit or AgNPs-PEG suspension in EW or LC at final concentrations of 0, 0.25, 0.5, and 1 μg/mL for LC and 0, 1.25, 2.5, and 5 μg/mL for EW, respectively. They were placed in single wells in a 48 well-plate (n = 12, triplicate) and incubated at 28.5 °C. Mortality was evaluated at 48 hpf, and hatchings were monitored at 96 hpf using a stereoscope (Nikon, SMZ18, Tokyo, Japan).

## 3. Results

### 3.1. Syntheses and Characterizations of AgNPs-Cit and AgNPs-PEG

**Figure 2:**
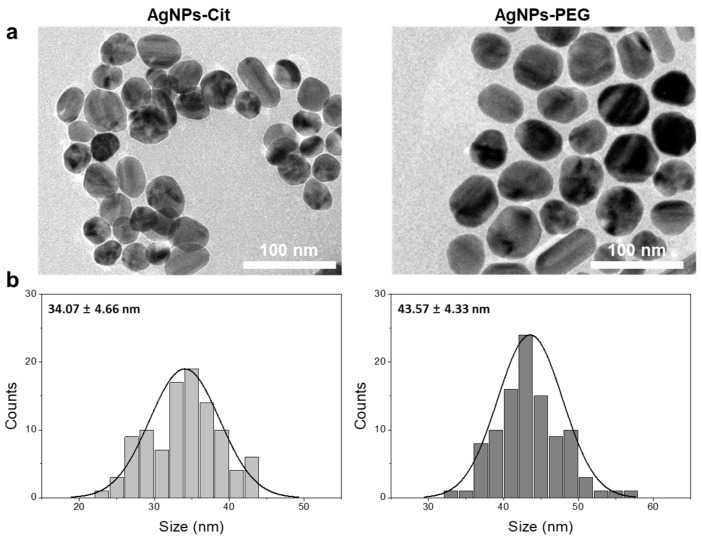
Characterizations of AgNPs-Cit and AgNPs-PEG. (**a**) Transmission electron microscopic (TEM) image and (**b**) size distribution.

The surface coating of nanoparticles with polymeric materials usually enhances nanoparticle stability in various solvents or media. Polymeric materials such as PEG, polyvinylpyrrolidone (PVP), and polyvinyl alcohol (PVA) have been widely used as coating materials for surface functionalization due to their biocompatibility. In this study, we functionalized AgNPs-Cit via pseudo-covalent binding between thiol termini of PEG and AgNPs to provide high stability to AgNPs in various ionic environments and reduce the toxicity of AgNPs by inhibiting ionization. In order to confirm the synthesis of the particles, AgNPs-Cit and AgNPs-PEG were analyzed using FE-TEM. The FE-TEM image and size distribution diagram in Figure 2a,b show that as-synthesized AgNPs-Cit were slightly irregular but mainly round, with average diameter of 34.1 ± 4.7 nm. Meanwhile, the average diameter of AgNPs-PEG was 43.6 ± 4.3 nm. To observe the stability of two AgNPs, we measured the zeta potentials and the hydrodynamic diameters of AgNPs-Cit and AgNPs-PEG in three different media (i.e., distilled water (DW), EW, and LC). The zeta potentials of AgNPs-Cit dispersed in DW was −46.8 ± 1.5 eV. As AgNPs-Cit dispersed in the EW and LC media, the zeta potentials decreased to −10.8 ± 0.7 eV and −16.8 ± 1.6 eV, respectively. In the case of AgNPs-PEG, the zeta potentials in DW were −18.2 ± 0.7 eV. They decreased to −4.13 ± 0.3 eV and −5.98 ± 0.5 eV in EW and LC, respectively (Figure S1). In Figure S2, the hydrodynamic size of AgNPs-Cit dispersed in DW was stable with elapsed time. However, the hydrodynamic size of AgNPs-Cit increased 10 times (D_EW_ ~ 363 nm) at 2 d in EW, and it dramatically increased (D_LC_ ~4000 nm) in LC medium. In contrast, AgNPs-PEG diameters were slightly bigger than AgNPs-Cit diameters due to polymer coating, and their sizes were kept consistent regardless of the media type during period (D_DW_ ~ 45 nm, D_EW_ ~ 42 nm, and D_LC_ ~ 44 nm). This indicates that the covalently-coated AgNPs-PEG were highly stable and resistant to dissolution or agglomeration induced by ionic environment. 

### 3.2. Transformation Behaviors of AgNPs-Cit in Different Ionic Environments

**Figure 3:**
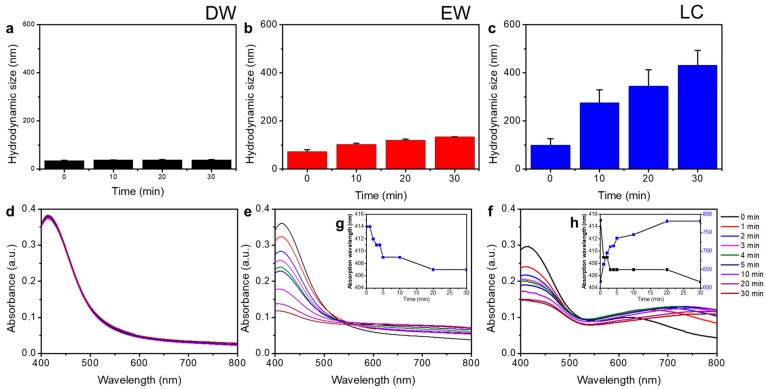
Physicochemical properties of AgNPs-Cit. (**a**–**c**) Hydrodynamic diameter and (**d**–**f**) absorbance of AgNPs-Cit were measured in distilled water (DW), E3 egg water (EW), and low chloride (LC) medium by time (0~30 min). AgNPs-Cit were dispersed at same concentration (5 µg/mL) in each medium. The inset graph of (**g**,**h**) indicate shifts of AgNPs-Cit absorbance in different media (DW, EW, and LC) with elapsed time (0~30 min).

To understand the behavior of AgNPs-Cit in different ionic environments, we measured the hydrodynamic size and absorption spectra of AgNPs-Cit for 30 min in each medium. In Figure 3a–c, the hydrodynamic size of AgNPs-Cit dispersed in DW showed no change during given time. However, the hydrodynamic size of AgNPs-Cit dispersed in EW slightly increased with time (D_EW_ 73.0 ± 6.8 nm ~ 133.4 ± 0.9 nm), and they grew rapidly as soon as AgNPs-Cit dispersed in LC (D_LC_ 99.2 ± 27 nm at 0 min toward 431.1 ± 62.2 nm at 30 min). AgNPs-Cit are colloidal metal nanoparticles and are typically yellow, because AgNPs have strong and broad optical absorption bands, which provide AgNPs with the properties of localized surface plasmon resonances (LSPR), and LSPRs are influenced by the size, shape, surface chemistry, and surrounding environments [19]. In Figure 3d, the absorption spectra of AgNPs in DW showed distinctive peak at 414 nm corresponding to the characteristic LSPR of AgNPs. However, the values of AgNPs-Cit absorption peaks in EW were decreased from 0.363 to 0.118 (Figure 3e), and peak positions were slightly blue-shift from 414 nm to 407 nm (Inset Figure 3g) by the time. This suggests that the concentrations and sizes of AgNPs-Cit decreased. In LC medium, the UV-Vis absorption peaks decreased from 0.297 to 0.148, and peak positions blue-shifted from 415 nm to 405 nm (Figure 3f). Simultaneously, an absorption peak appeared in the wavelength range between 618 nm and 781 nm. This seemed to be a result of a process known as the Ostwald ripening process, in which the size of nanoparticles becomes smaller and the size of larger nanoparticles becomes larger [20]. Due to the low chloride ion concentration in the LC medium, Ag^+^ ions seemed to be adsorbed to the surface of nearby AgNPs instead of being precipitated by reaction with chloride ions. This means that AgNPs-Cit, suspended in LC medium, consistently released Ag^+^ ions. Based on the results of the hydrodynamic size and LSPR change of AgNPs-Cit in different ionic environments, it was clear that the transformation behaviors of AgNPs were critically influenced by the concentration of chloride ion and the kind of dissolved electrolytes. This was confirmed by XPS spectra analysis (Figure S3). The distinctive Ag3d5 peaks at 367 eV and 374 eV were clearly seen in DW, and these peaks were weak in EW and disappeared in LC, assuming that they were caused by dissolved Ag by interaction with either high or low chloride in each medium. Meanwhile, the Cl 2p peak at 198 eV appeared in EW and LC not DW, indicating the formation of chlorocomplex in both EW and LC. 

### 3.3. Toxicities of AgNPs-Cit and AgNPs-PEG on Zebrafish Embryos

**Figure 4:**
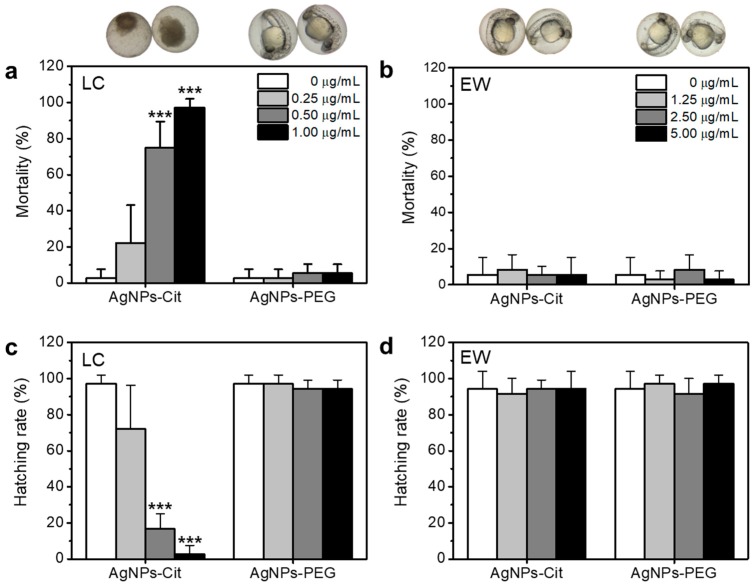
Mortality (**a**,**b**) and hatching rate (**c**,**d**) of AgNPs-Cit or AgNPs-PEG treated zebrafish embryos in different media (LC and EW). Each sample was treated at 4 hpf and incubated at 28 °C. Mortality was evaluated at 48 hpf, and hatching rate was observed at 96 hpf. All zebrafish embryos (n = 12, triplicate) were used in this study. The asterisk (***) indicates a significant difference between the treatment group and control (*p* < 0.001).

To evaluate and compare the toxicities of AgNPs in different ionic environments, AgNPs-Cit and AgNPs-PEG were assessed for toxicity using zebrafish embryos in individual test. AgNPs-Cit and AgNPs-PEG were treated on zebrafish embryos at 4 h post fertilization (hpf) incubated in EW and LC media, and the mortality and hatching rates at 48 hpf and 96 hpf were observed, respectively. As Figure 4a shows, the mortality of AgNPs-Cit in LC increased dose-dependently. However, AgNPs-PEG in LC medium showed low toxicity to the zebrafish embryo. Similarly, both AgNPs-Cit and AgNPs-PEG in EW displayed low toxicities, even at higher concentrations of AgNPs-Cit than LC (Figure 4b). AgNPs-PEG, stable particles in the ionic environment confirmed by hydrodynamic size and absorption spectrum measurements, showed very low toxicities in zebrafish embryos in both EW and LC. On the other hand, AgNPs-Cit caused serious toxicities in the zebrafish embryos, probably due to rapid dissolution in the LC. Ag^+^ ions released from AgNPs-Cit can inhibit the development of zebrafish embryos by binding to the thiol group of antioxidant proteins that includes glutathione (GSH), superoxide dismutase (SOD), and thioredoxin [21]. As a pretest, we confirmed the toxicities of Ag^+^ ions as a control experiment using silver nitrate (AgNO_3_) in Figure S4. Thus, zebrafish embryos at 4 hpf were significantly affected by AgNPs-Cit in LC. In case of AgNPs-Cit in EW, zebrafish embryos were much less affected by the existence of AgNPs-Cit, because Ag^+^ ions tend to be easily precipitated due to the existence of highly concentrated chloride ions. 

Hatching is one of the processes of zebrafish embryo development, and it usually occurs between 48 and 72 hpf. The hatching rate is a toxicity indicator of AgNPs-Cit and AgNPs-PEG, along with mortality. In Figure 4c,d hatching rates were observed for 96 hpf and showed opposite results compared with the mortality. Here, all non-hatched zebrafish embryos can be considered as non-survivors. It means that the early cell stage of embryos treated with AgNPs-Cit was significantly influenced by the survival of zebrafish embryo.

## 4. Discussion

In this study, we demonstrated that the toxicity of AgNPs is closely correlated to the dissolution behavior of AgNPs. In order to understand the transformation change of AgNPs, we analyzed physicochemical properties of AgNPs and conducted toxicity test using zebrafish embryos. Recent studies suggested that the Ag^+^ ions released from AgNPs played critical role to the acute toxicity of zebrafish embryo [11]. AgNPs exhibit different physicochemical properties depending on size, shape, surface chemistry, temperature, pH, and ionic strength. These factors induce different behaviors of aggregation, precipitation, or dissolution of AgNPs and have a significant effect on toxicity (Equations (1) and (2)). AgNPs, under oxygen rich environment, released Ag^+^ ions through oxidation reaction, resulting in the reaction with chloride ions to form AgCl(s). While aggregation or precipitation can reduce toxicity of AgNPs, dissolution can accelerate the toxicity of AgNPs due to the released Ag^+^ ions [22].

(1) $${\mathrm{Ag}}_{\left(s\right)}+\frac{1}{2}O_{2\left(\mathrm{aq}\right)}↔2{\mathrm{Ag}}^{+}+H_{2}O_{\left(l\right)}$$

(2) $${\mathrm{Ag}}^{+}{+\mathrm{Cl}}^{-}↔\mathrm{AgCl}\left(s\right)$$

The release of Ag^+^ ions is important for understanding the behavior of AgNP in different ionic environments. Zhang et al. suggested AgNPs ions release kinetics using Arrhenius equation (Equation (3)) [23]:

(3) $$\gamma_{{\mathrm{Ag}}^{+}}=\frac{3}{4}{\left(\frac{8\pi k_{B}T}{m_{B}}\right)}^{1/2}\rho^{-1}\exp\left(\frac{-E_{a}}{k_{B}T}\right)\left[\mathrm{Ag}\right]r^{-1}{\left[O_{2}\right]}^{0.5}{\left[H^{+}\right]}^{2}$$

in which $k_{B}$ is the Boltzmann constant (1.38 × 10^−23^ J·K^−1^), $m_{B}$ is the molecular weight of reactant *B* (g·mol^−1^), ρ is density of AgNPs, $E_{a}$ is the activation energy, and *T* is temperature (298 K). [Ag] is the mass-based concentration of AgNPs. [O_2_] and [H^+^] are the molar concentrations of oxygen and proton. r is the radius of AgNPs. This equation described that the Ag^+^ ions release depended on concentrations of AgNPs, O_2_, and H^+^ in inverse proportion to r. Peretyazhko et al. reported that dissolution kinetics of the AgNP correlated with size of nanoparticles and pH condition [20], and Li et al. showed the size of AgNPs increased by increasing NaCl concentration [24]. Based on the results in previous research, we hypothesized that the toxicity of AgNPs depends on the environment of AgNPs. For zebrafish embryo development, various electrolytes were dissolved in medium. The electrolytes increased ionic concentration and interacted with AgNPs. The increased ionic concentration leads to aggregation by decreased degree of electrical double layers repulsion. This can be confirmed by the result of decreased zeta potential in ionic environments such as EW and LC (Figure S1). It means that AgNPs can be aggregated due to decreased electrostatic energy barrier [20,23]. In Figure S2a and Figure 3a–c, AgNPs-Cit in LC medium showed less stable hydrodynamic size behavior than in EW over time due to higher concentrations of oxygen. Zhang et al. also showed random distribution of hydrodynamic size of AgNPs in the presence of dissolved oxygen [23]. The released Ag^+^ ion increases local ion concentration and causes a decrease in the sizes of AgNPs. Therefore, aggregation of AgNPs can be accelerated due to reduced electrostatic repulsion and increased surface energy. On the other hand, hydrodynamic sizes of AgNPs-PEG were observed to be stable in EW and LC media. This is because polymeric coating materials such as PEG or PVP can improve the stability of AgNPs via stabilizing effect based on steric repulsion between AgNPs [25,26,27].

Figure 3d–f showed absorbance of AgNPs. LSPR is a phenomenon occurring in the metal nanoparticles, not metal ions [28]. Absorbance of AgNPs can be described by Beer-Lambert law (Equation (4)) [29]:

(4) $$\mathrm{Absorbance}\left(A\right)=\log_{10}\frac{I_{0}}{I_{\lambda }}=\varepsilon cl$$

in which $I_{0}$ is the intensity of the incident light, and $I_{\lambda }$ is the intensity of the transmitted light. *ε* is the extinction coefficient, c is the concentration of the nanoparticles, and l is the path length of the light. Beer-Lambert law indicated that absorbance depends on the concentration of the particles. Figure 3e,f showed the absorbances of AgNPs decreased over time. Because AgNPs in EW and LC media were decomposed by dissolved oxygen, AgNPs concentrations decreased. Ionic environment critically influenced the physicochemical properties of AgNPs. It has also showed that the absorbances of AgNPs were decreased by dissolution [20,30]. Because PEG successfully protected AgNPs from dissolution or precipitation in the ionic environment, AgNPs-PEG showed stability over time. 

Figure 4 showed that toxicity of AgNPs-Cit and AgNPs-PEG depended on concentration, ionic environment, and surface coating. LC medium increased toxicity of AgNPs; however, EW medium and surface coating inhibited toxic effect. As Ag^+^ ion release rate can be accelerated by AgNPs concentration and dissolved oxygen (Equation (3)), AgNPs in LC medium increased toxicity by increased concentration (Figure 4a). Lee et al. showed that AgNPs toxicity depended on quantities of Ag^+^ ions and size [31]. Since zebrafish embryos at 4 hpf are cellular stages before tissue development, Ag^+^ ion causes reactive oxygen species (ROS) generation, deoxyribonucleic acid (DNA) damage, or mitochondria destruction during development. Thus, AgNPs in LC medium can have a highly adverse effect on zebrafish embryos. 

## 5. Conclusions

In summary, we demonstrated correlation between dissolution behavior and toxicity of AgNPs in different ionic environments. AgNPs can exert different toxic effects depending on the environment and the surface properties. In various environments, AgNPs can be precipitated, aggregated, and dissolved depending on ionic conditions. Based on the result of the dissolution behavior of AgNPs, we found that low concentration of chloride ion influenced on the rapid dissolution of Ag^+^ ion from AgNPs and resulted in toxicity of zebrafish embryos. Moreover, the surface functionalization of AgNPs inhibited the dissolution of Ag^+^ ion and reduced the toxicity in any ionic condition. This study will provide insight into nanomaterials’ behaviors in environments, as well as clues to minimizing their influence on organisms.

## Figures and Tables

**Table 1:** Composition of egg water (EW) and low chloride (LC) medium**.**

Component	EW (1X)	LC (1X)
NaCl	5 mM	0 mM
KCl	0.17 mM	0 mM
CaCl_2_	0.33 mM	0.024 mM
MgSO_4_	0.33 mM	0.791 mM
Na_2_HPO_4_	0 mM	0.254 mM
KH_2_PO_4_	0 mM	0.176 mM
NaHCO_3_	0 mM	3.095 mM

## Supplementary Materials

The following are available online at http://www.mdpi.com/2079-4991/8/9/652/s1, Figure S1: Zeta-potential, Figure S2: Hydrodynamic size, Figure S3: XPS analysis, Figure S4: Mortality of AgNO_3_-treated zebrafish embryos in LC medium.

## References

[1] Vance M.E., Kuiken T., Vejerano E.P., McGinnis S.P., Hochella M.F., Hull D.R. (2015). Nanotechnology in the real world: Redeveloping the nanomaterial consumer products inventory. Beilstein J. Nanotechnol..

[2] Beyene H.D., Werkneh A.A., Bezabh H.K., Ambaye T.G. (2017). Synthesis paradigm and applications of silver nanoparticles (AgNPs), a review. Sustain. Mater. Technol..

[3] De Matteis V., Cascione M., Toma C., Leporatti S. (2018). Silver Nanoparticles: Synthetic Routes, In Vitro Toxicity and Theranostic Applications for Cancer Disease. Nanomaterials.

[4] Le Ouay B., Stellacci F. (2015). Antibacterial activity of silver nanoparticles: A surface science insight. Nano Today.

[5] Furtado L.M., Norman B.C., Xenopoulos M.A., Frost P.C., Metcalfe C.D., Hintelmann H. (2015). Environmental Fate of Silver Nanoparticles in Boreal Lake Ecosystems. Environ. Sci. Technol..

[6] Blaser S.A., Scheringer M., MacLeod M., Hungerbühler K. (2008). Estimation of cumulative aquatic exposure and risk due to silver: Contribution of nano-functionalized plastics and textiles. Sci. Total Environ..

[7] Levard C., Hotze E.M., Lowry G.V., Brown G.E. (2012). Environmental transformations of silver nanoparticles: Impact on stability and toxicity. Environ. Sci. Technol..

[8] Liu J., Sonshine D.A., Shervani S., Hurt R.H. (2010). Controlled Release of Biologically Active Silver From Nanosilver Surfaces. ACS Nano.

[9] Levard C., Reinsch B.C., Michel F.M., Oumahi C., Lowry G.V., Brown G.E. (2011). Sulfidation processes of PVP-coated silver nanoparticles in aqueous solution: Impact on dissolution rate. Environ. Sci. Technol..

[10] Zhang W., Xiao B., Fang T. (2018). Chemical transformation of silver nanoparticles in aquatic environments: Mechanism, morphology and toxicity. Chemosphere.

[11] Groh K.J., Dalkvist T., Piccapietra F., Behra R., Suter M.J.F., Schirmer K. (2015). Critical influence of chloride ions on silver ion-mediated acute toxicity of silver nanoparticles to zebrafish embryos. Nanotoxicology.

[12] Cunningham S., Brennan-Fournet M.E., Ledwith D., Byrnes L., Joshi L. (2013). Effect of nanoparticle stabilization and physicochemical properties on exposure outcome: Acute toxicity of silver nanoparticle preparations in zebrafish (Danio rerio). Environ. Sci. Technol..

[13] Asharani P.V., Lianwu Y., Gong Z., Valiyaveettil S. (2011). Comparison of the toxicity of silver, gold and platinum nanoparticles in developing zebrafish embryos. Nanotoxicology.

[14] Yang X., Gondikas A.P., Marinakos S.M., Auffan M., Liu J., Hsu-Kim H., Meyer J.N. (2012). Mechanism of silver nanoparticle toxicity is dependent on dissolved silver and surface coating in caenorhabditis elegans. Environ. Sci. Technol..

[15] Panacek A., Prucek R., Safarova D., Dittrich M., Richtrova J., Benickova K., Zboril R., Kvitek L. (2011). Acute and Chronic Toxicity Effects of Silver Nanoparticles (NPs) on *Drosophila melanogaster*. Environ. Sci. Technol..

[16] Lin S., Lin S., Zhao Y., Nel A.E. (2013). Zebrafish: An in vivo model for nano EHS studies. Small.

[17] Chakraborty C., Sharma A.R., Sharma G., Lee S.S. (2016). Zebrafish: A complete animal model to enumerate the nanoparticle toxicity. J. Nanobiotechnology.

[18] Westerfield M. (2000). The Zebrafish Book. A Guide for the Laboratory Use of Zerbafish (Danio Rerio).

[19] Peng S., McMahon J.M., Schatz G.C., Gray S.K., Sun Y. (2010). Reversing the size-dependence of surface plasmon resonances. Proc. Natl. Acad. Sci. USA.

[20] Peretyazhko T.S., Zhang Q., Colvin V.L. (2014). Size-controlled dissolution of silver nanoparticles at neutral and acidic pH conditions: Kinetics and size changes. Environ. Sci. Technol..

[21] Riaz Ahmed K.B., Nagy A.M., Brown R.P., Zhang Q., Malghan S.G., Goering P.L. (2017). Silver nanoparticles: Significance of physicochemical properties and assay interference on the interpretation of in vitro cytotoxicity studies. Toxicol. Vitr..

[22] Peng C., Zhang W., Gao H., Li Y., Tong X., Li K., Zhu X., Wang Y., Chen Y. (2017). Behavior and Potential Impacts of Metal-Based Engineered Nanoparticles in Aquatic Environments. Nanomaterials.

[23] Zhang W., Yao Y., Sullivan N., Chen Y. (2011). Modeling the primary size effects of citrate-coated silver nanoparticles on their ion release kinetics. Environ. Sci. Technol..

[24] Li X., Lenhart J.J., Walker H.W. (2010). Dissolution-accompanied aggregation kinetics of silver nanoparticles. Langmuir.

[25] Chang W.C., Tai J.T., Wang H.F., Ho R.M., Hsiao T.C., Tsai D.H. (2016). Surface PEGylation of silver nanoparticles: Kinetics of simultaneous surface dissolution and molecular desorption. Langmuir.

[26] Huynh K.A., Chen K.L. (2011). Aggregation kinetics of citrate and polyvinylpyrrolidone coated silver nanoparticles in monovalent and divalent electrolyte solutions. Environ. Sci. Technol..

[27] Kittler S., Greulich C., Köller M., Epple M. (2009). Synthesis of PVP-coated Silver nanoparticles and their biological activity towards human mesenchymal stem cells. Materwiss. Werksttech..

[28] Kelly K.L., Coronado E., Zhao L.L., Schatz G. (2003). The Optical Properties of Metal Nanoparticles: The Influence of Size, Shape, and Dielectric Environment. J. Phys. Chem. B.

[29] Swinehart D.F. (1962). The Beer-Lambert Law. J. Chem. Educ..

[30] Kostigen Mumper C., Ostermeyer A.K., Semprini L., Radniecki T.S. (2013). Influence of ammonia on silver nanoparticle dissolution and toxicity to Nitrosomonas europaea. Chemosphere.

[31] Lee K.J., Browning L.M., Nallathamby P.D., Desai T., Cherukuri P.K., Xu X.H.N. (2012). In vivo quantitative study of sized-dependent transport and toxicity of single silver nanoparticles using zebrafish embryos. Chem. Res. Toxicol..

