# Supplementary material for: The Relationship between Dissolution Behavior and the Toxicity of Silver Nanoparticles on Zebrafish Embryos in Different Ionic Environments
Source: Nanomaterials (Basel). 2018 Aug 23;8(9):652. doi: 10.3390/nano8090652 (PMC6165318; doi:10.3390/nano8090652)
Supplement: Supplementary file 1 [file nanomaterials-08-00652-s001.pdf]

Supporting information

Relationship between dissolution behavior and toxicity of silver nanoparticles on zebrafish embryo in different ionic environments

[1] Korea Research Institute of Bioscience and Biotechnology, 125 Gwahak-ro, Yuseong-gu, Daejeon 34141, Korea; wang3026@kribb.re.kr (W.S.L.); alleles@kribb.re.kr (H.-J.C.); kangtaejoon@kribb.re.kr (T.K.); joody1006@naver.com (M.Y.K.); jsa1713@naver.com (Y.S.K.); jeongsoo@kribb.re.kr (J.-S.L.)
[2] KRIBB School, University of Science and Technology, Daejeon 34141, Korea
[3] Department of Chemistry, Korea Advanced Institute of Science and Technology, 291 Daehak-ro, Yuseong-gu, Daejeon 34141, Korea; kekgsm@kaist.ac.kr (E.K.); nanobio@kaist.ac.kr (B.K.)
[4] Advanced Instrumentation Institute, Korea Research Institute of Standards and Science, 267 Gajeong-ro, Yuseong-gu, Daejeon 34113, Korea; nwsong@kriss.re.kr
[5] Dementia DTC R&D Convergence Program, Korea Institute of Science and Technology, 5 Hwarang-ro, 14-gil, Seongbuk-gu, Seoul 02792, Korea
* Correspondence: jyjeong@kribb.re.kr; Tel.: +82-42-879-8454
† These authors contributed equally to this work.

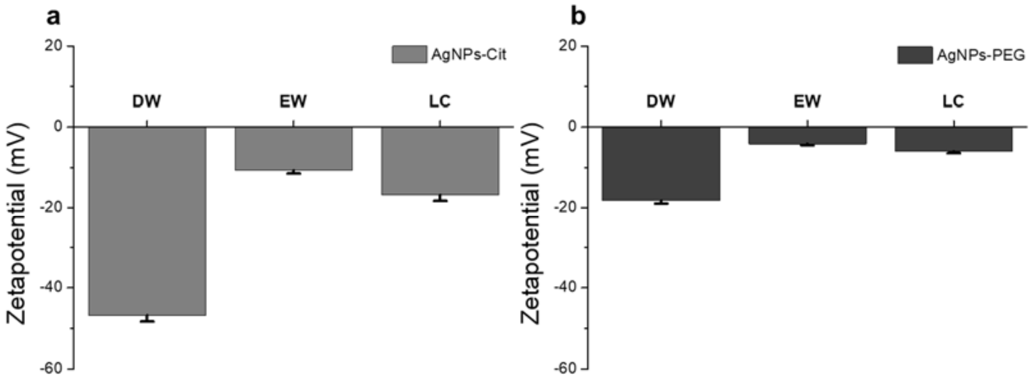

Figure S1. Zeta potential of AgNPs-Cit (**a**) and AgNPs-PEG in distilled water (DW), E3 egg water (EW), and low chloride (LC) medium. The particles were consistently dispersed at same concentration (5 μg/mL) in each medium.

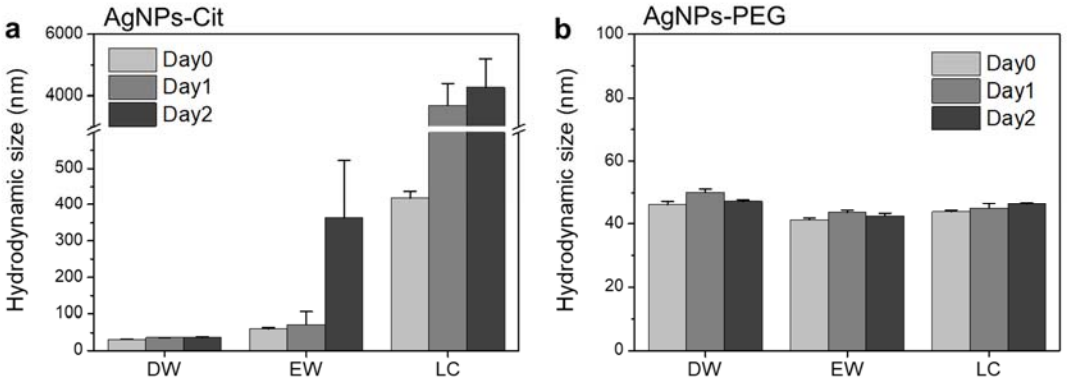

Figure S2. Hydrodynamic size of citrate-stabilized (**a**) AgNPs-Cit and (**b**) AgNPs-PEG in DW, EW, and LC medium. The particles were consistently dispersed at same concentration (5 μg/mL) in each medium.

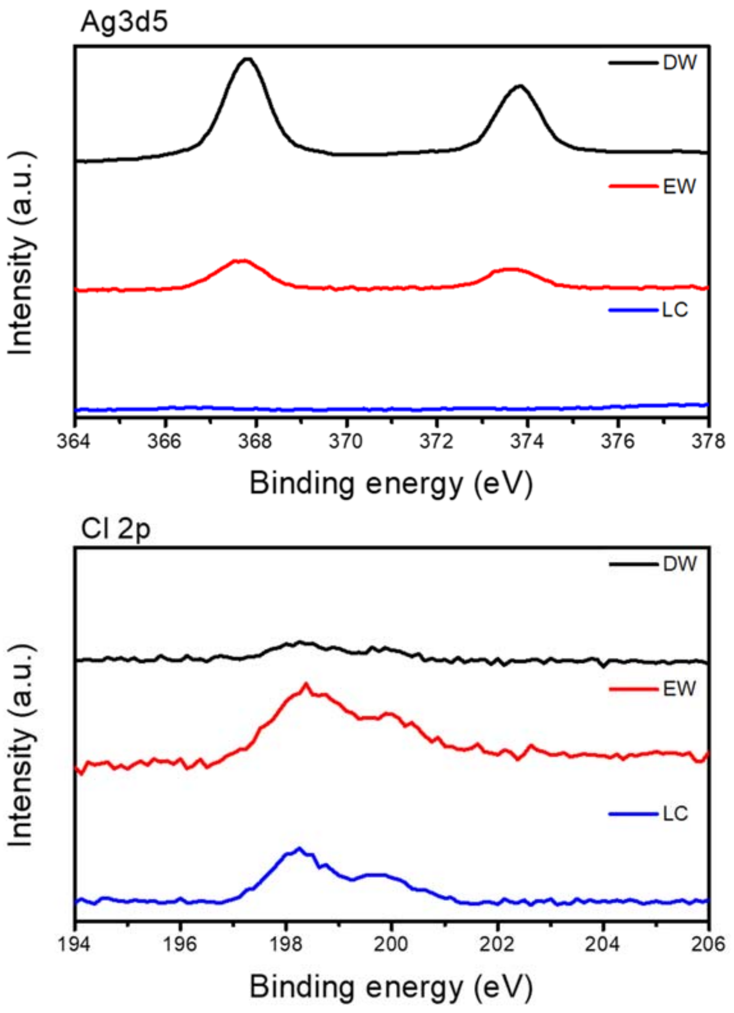

Figure S3. XPS analysis of AgNPs-Cit in different medium (DW, EW, and LC) after 1 day.

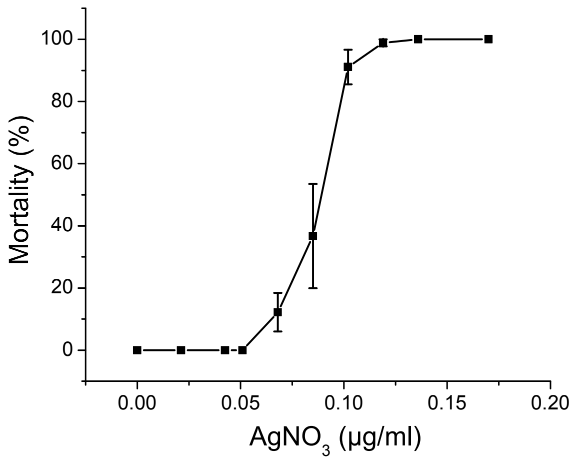

Figure S4. Mortality of $AgNO_3$ –treated zebrafish embryos in LC medium.
